# Adoption of App-Based Peer Support by Canadian Health Care Providers: Mixed Methods Organizational Implementation Study

**DOI:** 10.2196/85658

**Published:** 2026-08-05

**Authors:** Sandra Moll, Melissa Parker, Peter Smith, Edward Sykes

**Affiliations:** 1 Department of Occupational Therapy, Faculty of Health Sciences, McMaster University, 1400 Main St. West, Hamilton, ON, L8S 1C7, Canada, 1 9054672155; 2 Institute for Work and Health, Toronto, ON, Canada; 3 School of Computer Science, College of Computational, Mathematical and Physical Sciences, University of Guelph, Guelph, ON, Canada

**Keywords:** implementation science, mental health, technology, workplace, mhealth, mobile health

## Abstract

**Background:**

There is an urgent and critical need to support the mental health of health care providers, given high rates of stress and burnout. Although the issues are complex, digital access to information and support can help address the needs, as technology can facilitate on-demand links to private, customized resources, including peer support. Beyond Silence (McMaster University) is an evidence-informed mobile health platform co-designed with health care workers and grounded in prior evidence that mental health literacy and peer support can reduce stigma and facilitate earlier help-seeking.

**Objective:**

This study aimed to (1) explore how health care workers across diverse health care settings use the app and (2) identify opportunities and barriers to implementation.

**Methods:**

A multiple-case study framework, informed by the Consolidated Framework for Implementation Research (CFIR), was applied to capture 4 months of implementation across a purposive sample of 7 diverse Canadian health care organizations. Implementation within each organization was led by designated organizational champions who leveraged existing communication channels and standardized promotional materials to invite employees to voluntarily download and use the app. Implementation outcomes were assessed using app analytics (downloads and feature use) and semistructured baseline and follow-up interviews with organizational champions to explore contextual influences on uptake.

**Results:**

Approximately 1066 employees downloaded the app over the 4-month period, ranging from <2% to >45% of employees across the 7 organizations. Interviews with 28 organizational champions noted that there was good leadership support for the technology, aligning with their mission to address employee mental health. Barriers to use, however, included workplace culture surrounding mental health and help-seeking, lack of awareness about when and how to use the app, and infrastructure-related challenges, such as limited time and a lack of private spaces to download and use the technology.

**Conclusions:**

Effective implementation is a precondition for positive outcomes; therefore, strategies are needed to optimize technology implementation. Recommendations include evaluating organizational readiness, building mental health literacy, creating a multimodal communication and implementation plan, addressing technology requirements, and embedding the technology into organizational policies and practices. This study highlights key challenges in the implementation of the Beyond Silence peer support platform for health care workers, including slow adoption linked to mental health stigma, competing demands, and limited frontline engagement. Addressing these barriers will require innovative, trust-building strategies to support meaningful uptake and sustained use.

## Introduction

The mental health of health care providers demands immediate and sustained support. Health care workers experience elevated rates of stress and burnout, which have been exacerbated by the COVID-19 pandemic [1]. Sustained psychological burden and high workloads have led to a high rate of workforce attrition, leading to a human resource crisis that may take years to resolve [2]. Issues facing health care workers are complex and can range from high workloads to exposure to workplace violence and/or potentially traumatic situations [1]. For example, many clinicians feel isolated and unsupported by their workplace [3,4].

A growing body of literature underlines the value of peer support for health care workers in mitigating the impact of stress and posttraumatic stress disorders (PTSD) [5,6]. Peers understand the unique context of health care work and can therefore be a trusted source of support. Access to peer support, however, can be challenging in some workplace contexts, particularly if there is a negative workplace culture or resource and time pressures that make it difficult to reach out. E–mental health interventions are gaining recognition as a strategy to enable mental health support across different contexts, including the workplace [7]. Mobile technology can provide accessible, on-demand information and links to support and has the potential to reach a wide range of users, including those in underresourced settings [7,8]. Access to peer support via mobile technology can also be a cost-effective first-line approach and conduit for professional support within a stepped model of care [9].

Evidence for the effectiveness of mobile health (mHealth) apps has been noted through systematic reviews and meta-analyses, including evidence for reducing symptoms related to common mental health disorders such as anxiety and depression both in the general population and clinical populations [8,10]. In addition to studies in the general population, there are a growing number of studies examining the impact of digital mental health interventions in workplace settings [11]. One systematic review and meta-analysis of mHealth apps implemented in workplace settings found mixed evidence for improving mental health symptoms with poor sustainability of the effects over time [12]. An umbrella review was published in 2025 of 14 systematic literature reviews of the effectiveness of digital mental health interventions in the workplace (9 systematic reviews and 5 meta-analyses) [13]. The review noted growing evidence of the small to moderate impact of digital tools on reducing depression, anxiety, and perceived stress.

Although these studies highlight the potential of mHealth apps, outcomes remain variable across and within studies, and gaps persist in our understanding of implementation over time and across contexts [13]. A review of eHealth interventions for health care professionals, for example, highlighted inconsistencies in outcomes and limited implementation data [14]. Implementation science frameworks such as the Nonadoption, Abandonment, Scale-up, Spread, and Sustainability (NASSS) model of health and care technology highlight the challenges associated with consistent and sustained engagement with mHealth technology, grounded in an appreciation of the various complexities of implementation [15]. The Consolidated Framework for Implementation Research (CFIR) also notes the importance of understanding different domains of implementation, considering how the context and process of implementation shape adoption [16]. There remains a need not only to assess the impact of technologies but also to understand the factors influencing uptake and implementation, and how these evolve over time. It is not enough to understand whether an app is designed well; it is important to systematically explore how it is adopted in a real-world context [7,8,12]. This study aimed to (1) examine the adoption of Beyond Silence (McMaster University), an mHealth peer support platform designed by and for health care employees across 7 diverse Canadian health care organizations, and (2) identify and describe perceived organizational facilitators and barriers to its implementation both within and across these settings.

## Methods

### Study Design

A mixed methods implementation case study design [17] was adopted to explore implementation and uptake of the Beyond Silence platform across 7 health care organizations. A case study approach provides an effective way to study contemporary phenomena within a real-life context, particularly when it is difficult to separate the case from the context and can lead to new explanations that consider the complexities of place and time [18]. Exploring multiple case study sites enabled cross-case comparison across different workplace contexts. A multistage mixed methods framework was adopted, starting with qualitative interviews to explore the perceptions of organizational champions regarding the context for app implementation [19]. This was followed by the collection of quantitative app usage data. Finally, follow-up interviews were conducted with the organizational champions, during which they were asked about the individual and contextual forces that shaped trends in app usage within the workplace context [20]. Data collection and analysis were informed by CFIR, one of the most commonly used frameworks to assess contextual factors affecting real-world implementation [21].

### Ethical Considerations

Ethics approval was obtained through the Hamilton Integrated Research Ethics Board (HiREB number 14740), and all participants provided written consent to participate. In order to maintain privacy and confidentiality, the names of participating organizations and organizational champions are disguised. All app usage data are reported in aggregate; app users were anonymous. Organizational champions received a CAD $100 (US $70.95 as of July 24, 2026) gift card honorarium for participating in each champion interview. Peer supporters in each organization were not paid for their role; however, external peer support providers were provided a stipend of $55 per 3-hour shift. Peer support providers who participated in the focus group discussions were invited to enter a draw for one of 2 CAD $50 (US $36.50) gift cards of their choice.

An advisory team provided input throughout the project, including 4 academic leaders in workplace mental health, mHealth technology, and trauma-informed care, and 6 knowledge user partners representing national associations of nurses, personal support workers (PSWs), social workers, and long-term care workers, a national mental health service provider, and a provincial health and safety association. The team met 3‐4 times a year to provide input at key stages in the project and to facilitate communication within their extensive networks.

### Intervention

**Figure 1.**
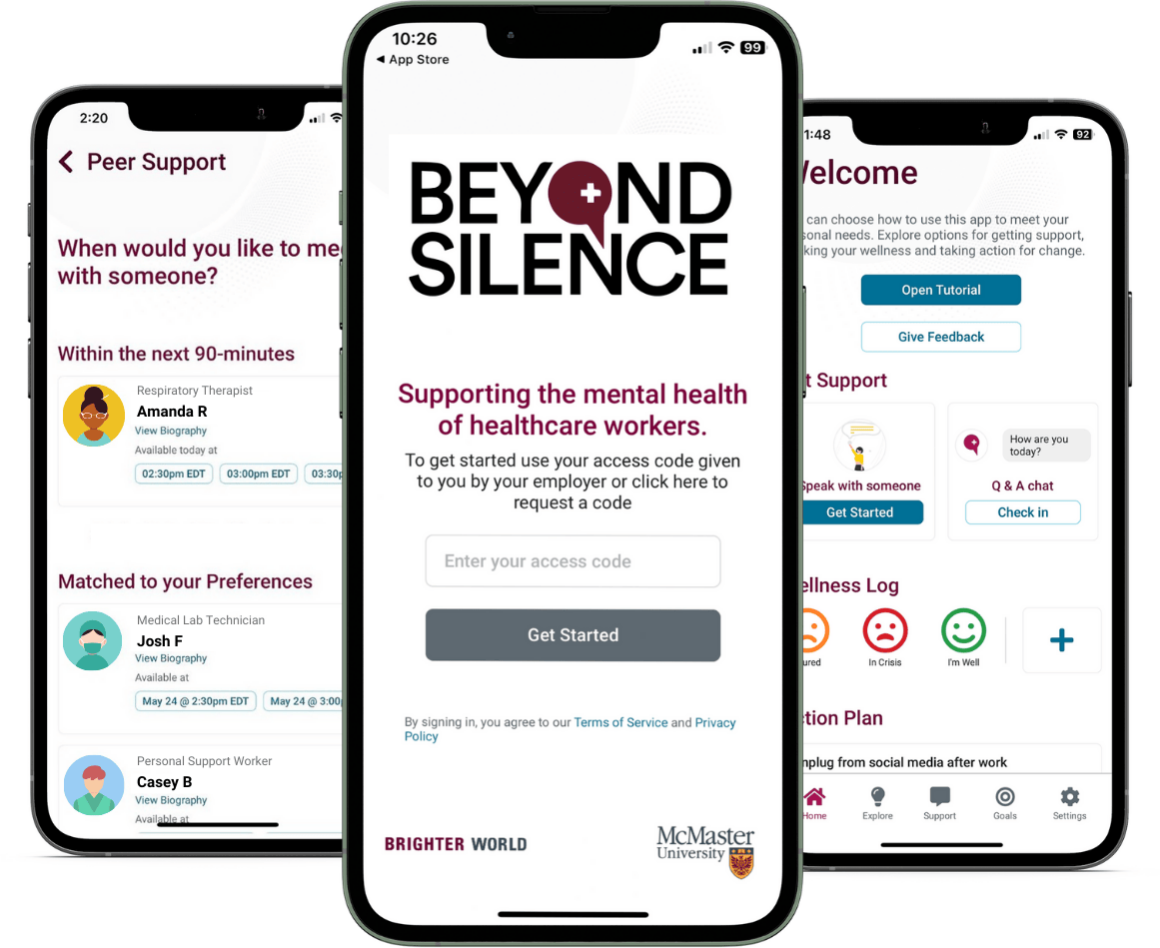
Screenshots of the Beyond Silence mobile app for frontline workers.

Beyond Silence (see Figure 1) is a digital platform (including a mobile app for frontline health care workers and an organizational portal for peer supporters and organizational champions) designed to provide accessible, anonymous, and peer-led mental health support to Canadian health care workers. It builds on a decade of successful research collaborations with health care stakeholders to identify and address their unique mental health and support needs [19,20]. Development was informed by findings from a randomized controlled trial of an in-person workplace mental health training program with over 200 health care workers [21]. The in-person training was effective but resource-intensive and not accessible to all workers; therefore, an mHealth platform was explored as an alternative approach.

Development of the Beyond Silence platform (2017‐2019) was supported by a Canadian Institute of Health Research (CIHR) development grant, and informed by the Knowledge-to-Action Framework, a structured approach for knowledge creation and a 7-phase cycle that moves knowledge into practice [22]. In addition, best practice principles of mHealth design were considered, including engagement (customization and interactivity), functionality (ease of use), aesthetics, information quality, and data security [23]. Additional funding (2022‐2024) from the Public Health Agency of Canada (PHAC) allowed for additional app enhancements. Core functionalities of the app for frontline workers include (1) access to evidence-informed information customized for health care workers (articles, infographics, video, and audio clips) on a range of topics from mindfulness to moral injury; (2) links to self-management tools, including a wellness toolbox, wellness check-ins, and goal-setting feature; and (3) private, secure links via text or phone to a range of health care providers trained to offer peer support. App users can browse the biographies of the available peer supporters within and outside their organization, choose how they want to connect (phone or text), click on who they want to connect, and the time at which they want to connect (either within the next 90 minutes or at a scheduled time that their preferred peer is available). A notification is then sent to the identified peer regarding the anonymous request for support and a secure, private connection is made through the app for the health care worker seeking support.

In addition to the app for frontline health care workers, the platform includes an interconnected web-based portal for trained peer support providers to respond to requests for support. The online portal provides space for peer supporters to upload their pictures and short biographies (visible to the app users), as well as sign up for 3-hour shifts, respond to peer support requests (by text or phone), record digital notes on their encounters, access resources to support their work (eg, crisis protocols and contact details for relevant resources), and connect with a clinical supervisor and/or other peer supporters. Although the peer support providers were visible to frontline app users, the person accessing support was anonymous (digital ID number). A total of 156 health care professionals were trained to offer peer support in the app over the course of the study, including 88 from participating organizations and 68 central peer supporters who were external to the participating organizations and accessible to all. Details of the peer support training are reported in a separate paper [24].

The Beyond Silence platform also included an administrative portal where organizational champions could upload local resources, manage their internal peer support provider list, send notifications, and track overall patterns of app use. It is important to note that the app was available through app stores, but an organizational code was required to download the app, since the app is customized for each organization (including local resources and peer support access). As noted above, there was an onboarding process for the participating organizations and peer supporters, which included training on risk management and data security protocols.

### Organization Recruitment

Health care organizations were recruited through established networks of the project advisory team. Interested organizations were asked to complete an application form to assist in screening for diversity across size, geographic location, workplace setting, and type of health care service. To be eligible to participate, organizations required: (1) a project agreement letter signed by organizational leaders, (2) at least one organizational champion designated to liaise with the research team, and (3) at least 2‐3 internal staff members who agreed to be trained peer support providers (numbers varied based on size and resources available within the organization).

The goal was to recruit 6‐8 organizations to explore how implementation varied across workplace contexts. Purposive sampling was conducted to ensure diversity in size (small/medium/large), geographical location, setting (rural/remote and urban), type of service (long-term care, acute care, tertiary care, and primary care), and at least 1 organization where employees spoke French (since the app is available in both French and English). A total of 22 organizations expressed interest in the study; however, 14 of these organizations did not have the capacity to participate. For organizations that declined to participate, the primary reason for declining was documented (typically due to limited resources to support the app implementation and evaluation).

Implementation setting and scope A champion-led, organization-embedded implementation strategy was used to introduce Beyond Silence within each participating organization. Champions were individuals representing participating organizations who approached us to be involved in the study. They were responsible for liaising with the research team, communicating about the app to employees, and acting as the contact point for any questions or concerns among staff. Each site designated one or more organizational champions to serve as local implementation leads and primary points of contact.

Champions represented a range of organizational roles, including senior leaders, health and safety coordinators, and frontline clinicians, including 20 women and 6 men in both clinical (n*=*13) and nonclinical (n=13) positions. As noted in Table 1, there were typically 1‐3 champions in each organization who agreed to communicate with employees about the app and to be interviewed about their insights at the outset and conclusion of the project. One large multisite organization had 11 champions volunteer to participate in the study.

**Table 1.** Participating health care organizations.

Organization	Employees, n	Dates	Champions, n	Trained providers, n
1. Urban community care (nurses)	600	November 2022-March 2023	1	3
2. National professional association	13,000		4	12
2A. Department in urban hospital		November 2022-March 2023		
2B. Nonclinical workers		November 2022-March 2023		
2C. National association		August 2023-December 2023		
3. Northern Ontario Health Centre	769	November 2022-March 2023	2	9
4. Urban community care (PSWs^a^)	866	November 2023-March 2024	2	6
5. Rural community care	55	November 2023-March 2024	2	3
6. Large urban hospital	12,463	November 2023-March 2024	12	19
7. National health service	5000	February 2024-June 2024	4	36
Total (all organizations)	32,753	November 2022-June 2024	27	88

a PSW: personal support worker.

The target population for implementation was determined by each organization based on local capacity and readiness. Most included all employees, but 3 organizations chose a specific subgroup (Organization 1–nurses, Organization 2A–laboratory technicians, and Organization 4–PSWs). Recruitment relied on existing organizational communication channels and included distribution of standardized promotional materials (posters, email scripts, brochures, and branded materials) containing information about the app, a QR code for download, and a unique organizational access code. Employees were invited to voluntarily download and use the app for the implementation period.

Implementation included organizational onboarding, app deployment to frontline staff, and integration of trained peer support providers accessible through the platform. Uptake, engagement, and variability were tracked across organizational contexts over a 4-month period. Guided by an implementation framework (CFIR), contextual factors, resource requirements, and implementation processes were examined to inform scalability and sustainability.

### Data Collection and Outcomes

Data collection focused on prespecified implementation outcomes and used a mixed methods approach combining app usage analytics and qualitative interviews with organizational champions. Evaluation focused on reach, adoption, implementation, and patterns of use rather than effectiveness or health impact.

Quantitative implementation metrics were assessed using real-time, aggregate app analytics captured through the administrative portal. App users remained anonymous and were identified only through a unique user ID. Metrics were selected to characterize adoption and engagement and included the number and proportion of employees who downloaded the app, the number of times the app was opened, mean number of app opens per user, and frequency of use of key features, including content views, goals set, wellness check-ins, and peer support appointments booked. Additional descriptive data captured peer support outreach modality (text or call) and choice of peer supporter. App use data were examined monthly within each organization to describe trends over the 4-month implementation period and to support cross-case comparison; inferential analyses were not conducted.

An update to the app occurred during which onboarding questions were added, asking app users to respond to optional demographic questions regarding age, gender, years of professional experience, and health care role (clinical/nonclinical). Given the timing of this change, only 5 of the 7 organizations included this demographic data. As such, demographic data were not collected or reported for organizations 1, 2A, 2B, and 3.

The study is registered on ClinicalTrials.gov (NCT05514093) as an implementation and evaluation study of a mHealth intervention designed to support the mental health of health care workers in Canada. However, while multiple mental health outcome measures (including standardized pre-post surveys of depression, anxiety, PTSD, burnout, and help-seeking behavior) were included in the study protocol, survey response rates at follow-up were too low to permit meaningful quantitative analysis. As a result, the mental health survey data are not reported, and no conclusions can be drawn regarding changes in mental health outcomes based on these measures.

### Qualitative Implementation Data

Qualitative data were collected to explore contextual factors influencing implementation. Interviews with the organizational champions included baseline interviews conducted prior to app implementation (n=19 interviews with 27 individuals) and follow-up interviews conducted at least 3‐4 months following implementation (n=18 interviews with 26 individuals). Interviews were conducted virtually, audio-recorded, and transcribed verbatim, with durations of approximately 60‐90 minutes. Some champions were interviewed individually, but others were interviewed in pairs or groups of 3. Interview guides with the champions were informed by the five dimensions of the CFIR, including questions exploring (1) perceptions of the intervention (characteristics of the app), (2) the outer setting (eg, societal context and policies), (3) the inner setting (eg, organizational culture and access to resources), (4) individuals (role of leaders, peer supporters, and characteristics of frontline workers), and (5) the implementation process [16]. Specific questions within each domain were developed using the CFIR interview guide tool. Since organizational champions were embedded within the organization, their insights enabled identification of critical factors that shaped uptake of the technology.

No predetermined numerical targets for adoption or engagement were specified. Given the exploratory nature of the implementation and the lack of established benchmarks for uptake of this type of workplace mental health and peer support app in comparable health care settings, the study aimed to descriptively characterize implementation outcomes and variability across organizations rather than assess performance against predefined thresholds.

### Analysis

Descriptive data regarding app use included monthly reporting within each organization to examine trends in downloads and use of the different features. Cross-case comparisons were also conducted to review similarities and differences in usage patterns across organizations, but inferential statistics were not calculated.

Organizational champion interview data were transcribed and coded, with initial coding categories informed by the CFIR. Coding was an iterative process that began with reviewing transcripts, categorizing them by the 5 CFIR domains (deductively), and then inductively identifying the key issues within each domain. At least 2 team members coded each transcript, initially coding 2‐3 transcripts independently, then meeting together to compare codes and refine the codebook to ensure consistency. A series of team meetings was held to discuss emerging analytic ideas and revise the codebook to capture key facilitators or barriers to implementation across organizational contexts. Strength and valence ratings of the key forces within each organization were conducted in order to facilitate cross-case comparison [24]. Identification of key forces represented comments across multiple organizations, with quotes selected to illustrate, considering representation across all participants. Dedoose (SocioCultural Research Consultants, LLC) software [25] was used to manage the qualitative interview data. The qualitative data were used to explain the app use trends and facilitate actionable insights to optimize implementation.

Strategies to ensure methodological rigor included triangulation of data sources (app use metrics and interviews with multiple champions over different time points in each organization). Triangulation strengthened in-depth understanding of the organizational context and implementation forces within and across organizations that shaped app use, leading to data saturation across categories and understanding of the contextual forces shaping objective patterns of app use. In order to ensure coding consistency, biweekly meetings were held with the research team to review the coding and to engage in peer debriefing. Meetings between coders were scheduled to calibrate approaches and refine definitions in the codebook until there was at least 85% coding consistency. Reflexive analysis was strengthened through analytic memos that were discussed in the peer debriefing meetings. CFIR was used as a sensitizing framework, but the iterative, inductive analysis process led to nuanced understanding of the key issues shared by participants. Member checking was conducted through sharing preliminary analyses with champions via draft organizational reports, then exploring their reflections on the analysis in follow-up interviews.

## Results

### Overview

The study findings are presented in three parts: (1) an overview of the participating organizations and app users, (2) descriptive metrics regarding patterns of app use, and (3) qualitative analysis of champion interview data regarding factors shaping app use (informed by the CFIR framework), considering the social and organizational context (outer and inner setting), response to the app innovation, influence of organizational stakeholders, and impact of the implementation strategies.

### Participating Organizations

A total of 7 diverse health care organizations participated in the study, joining at different time points. As outlined in Table 1, 2 of the organizations were large (over 10,000 employees), and one was small (n=55 employees). Organization 2 was a large professional association that engaged in the study at different time points, initially with a subgroup of workers (2A/2B) and then later as the full organization (2C). Their participants were members of the association (paying membership dues), rather than employees, and the app was offered as a member benefit. Organizations 1 and 4 chose to focus on a subgroup of health care providers within the organization (nurses and PSWs, respectively), but other organizations provided access to all employees (clinical and nonclinical). Organizations 3, 6, and 7 were multisite facilities, and organizations 1 and 4 provided community care with no on-site services. The number of champions and peer supporters varied across sites. As such, there were various implementation contexts for implementation. The app for frontline workers was made available to a total of 32,753 health care workers across these organizations.

Demographic data were collected from app users in organizations 2C, 4, 5, 6, and 7 via onboarding demographic questions that were added in June 2023. As noted in Table 2, app users in these organizations (n=840) included 77% (n=650) who identified as women, 21% (n=173) as men, and 2% (n=17) as nonbinary or chose not to disclose. Just over half of participants were between the ages of 30 and 49 (n=458, 55%) years. In terms of professional experience, 41% (n=348) had ≥16 years, followed by 15% (n=129) with 6‐10 years. Most participants held clinical roles (n=505, 60%), while 24% (n=205) were nonclinical.

**Table 2.** Characteristics of app users (in organizations 2C, 4, 5, 6, and 7).

Characteristic	Participants
Age (years), n (%)	
18‐29	103 (12)
30‐39	217 (26)
40‐49	241 (29)
50‐59	204 (24)
60‐69	47 (6)
Undisclosed	28 (3)
Gender, n (%)	
Man	173 (21)
Woman	650 (77)
Nonbinary or undisclosed	17 (2)
Experience (years), n (%)	
<2	101 (12)
2‐5	108 (13)
6‐10	219 (15)
11‐15	114 (14)
≥16	348 (41)
Undisclosed	40 (5)
Role, n (%)	
Clinical	505 (60)
Nonclinical	205 (24)
Undisclosed	130 (16)

### App Engagement Metrics

A total of 1066 employees downloaded the app across all organizations over the 4-month implementation trial. As noted in Table 3, the number and proportion of downloads within each organization ranged from <2% (n=108) in the largest organization to >45% (n=25) in the smallest organization. The mean download rate across organizations was approximately 11.6% (SD 15.2) of employees, but the overall downloads represented only 3% of the total number of eligible participants.

**Table 3.** App user downloads and feature use by organization.

Organization	Downloads, n (%)	App opens	Mean opens/ user	Content views, n (%)	Goals set, n (%)	Wellness check-ins, n (%)	Appointments booked (n)
1	38 (6)	127	3.34	32 (25)	2 (2)	13 (10)	2
2	112 (2)	457	4.89	154 (34)	38 (8)	79 (17)	49
3	84 (11)	273	3.25	38 (14)	24 (9)	43 (16)	4
4	73 (8)	157	2.15	29 (18)	6 (4)	23 (15)	4
5	25 (45)	85	3.40	63 (74)	19 (22)	19 (22)	0
6	468 (4)	1324	2.83	323 (24)	73 (6)	220 (17)	16
7	266 (5)	704	2.65	140 (20)	39 (6)	95 (13)	21
Total	1066 (3.24)	3127	—^a^	779	201	492	94
Average, mean (SD)	11.6 (15.2)	—	3.22	111	29	70	10

a Not applicable.

User engagement, measured by app opens and feature use, also varied across organizations. As noted in Table 3, the app was opened 3127 times over the course of the study, ranging from a total of 85 opens (45% of employees) in the smallest organization (Organization 5) to 1324 opens (4% of employees) in the second largest organization (Organization 6). Each downloaded app was opened a mean of 3.22 times (range from 2.1 to 4.9).

Given the sample size, normal approximation methods were used to calculate 95% CIs for proportions. A total of 309 app users viewed at least one content item, representing 30% of users (95% CI 27.2%‐32.9%). When viewing the content, users could provide a rating from 1 to 3 stars. There were 105 user-submitted content ratings, with a mean rating of 2.58 (SD 0.68) out of 3, corresponding to 86% of the maximum possible score. The most frequently accessed content by app users was an article titled “6 Mindfulness Exercises,” which received more than twice as many views (n=92) as the next most accessed item (n=35).

Engagement with other app features included (1) wellness check-ins initiated by 25% of app users (95% CI 22.3%‐27.7%), representing between 10% and 22% of the app openings; (2) goal setting initiated by 13% of app users (95% CI 11.0%‐15.2%); and (3) peer support outreach was accessed 94 times (56 calls and 38 texts) by 40 unique app users, with a wide range from 0 (in the smallest organization) to 49 (in the largest organization). Appointments were booked with 38 different peer support providers.

### Analysis of Forces Shaping App Engagement

#### Overview

Data from 19 baseline and 18 follow-up interviews with organizational champions (n=37 in total) informed the analysis of factors shaping patterns of app use. Informed by dimensions of the CFIR framework (outer/external context, inner/organizational context, the app innovation, the individuals’ role/characteristics, and the implementation process), we will highlight key facilitators and barriers to engagement noted by the organizational champions, followed by an in-depth analysis of implementation strategies within and across organizations. See Table 4 for a summary of the findings organized by the CFIR dimensions. Participant quotations to illustrate each issue are identified by their organizational affiliation.

**Table 4.** Summary of facilitators and barriers to app use.

CFIR dimension	Facilitator	Barrier
Outer context	High mental health needs as a result of system/pandemic pressures	High fatigue, burnout, and mistrust of new initiatives as a result of system/pandemic pressures
Inner context	Alignment with organizational mission to support staff Complements other wellness initiatives	Stigma associated with the need for mental health support Culture of skepticism/mistrust Not able to use phones at work Unstable internet No time or private space to call
Innovation characteristics	Easy to use, accessible, relevant content, choice re: when/how to access support, anonymous and private connection to support	Time needed to download
Individual roles and characteristics	Commitment from organization champions and leaders Trained peer supporters (local and external)	Health care worker reluctance to reach out for support Limited digital literacy
Implementation process	In-person connections Leadership endorsement Messaging from trusted organization champions Ongoing, strategic communication	Passive promotion (email and posters) not engaging In-person events don’t reach everyone (shift work and offsite) Employer-endorsed approach can be met with suspicion Lack of proactive follow-up Lack of integration with other wellness initiatives

#### Opportunities and Facilitators to Uptake

One consistent finding was that the external context of health care has created the need for mental health support. Many champions talked about the unique challenges and pressures faced by frontline health care workers. Even though the work context ranged from acute hospitals to community rehabilitation, and from large urban centers to small rural settings, almost all champions described challenges related to workload that were exacerbated by the COVID-19 pandemic, and how this elevated the level of fatigue and staff burnout. As one champion explained, "right now, we’re seeing levels of burnout and a bit of jadedness that’s come from being in this grind of the pandemic for quite a long time*”* (Organization 2). Similarly, another champion noted, *“*Health care is still reeling [from] the effects of COVID…there are staffing pressures and fatigue,*”* underlining the urgent need for support, explaining that *“*the building is burning as we’re trying to build the new fire station” (Organization 6). As a result, organizations were acutely aware of the significant pressures on staff members and the urgent need to support their frontline workers.

Introducing a tool like the Beyond Silence platform also aligned with the inner context or mission of most participating organizations to support employee mental health. Organizational planning in all organizations included a key focus on employee mental health and included support from the organizational leadership team. Champions explained that the app for frontline workers complemented existing wellness strategies, noting that peer support and digital tools were complementary or even natural extensions of their ongoing psychological health and mental wellness efforts. As noted below, one champion explained that the app was a catalyst for innovation and expansion of their wellness services:

*It came at a very good time because our psychological health and wellness initiatives were occurring. And actually, having the app helped us broaden the people involved in our peer support efforts…. it also gave us momentum to do some creative things*

[Organization 3]

There were also many characteristics of the app that were valued. Participants valued the relevance of the content, with customization to the unique context of health care. It was noted that the app was “easy to use... helpful, interesting articles, appropriate… relevant” (Organization 1). One of the participants explained:

*the fact that it’s geared specifically for health care providers, I think was really important for us. Anytime we’re looking at providing a new product or service to our members, making sure that … it’s very specific to their role. And so that was really appealing*

[Organization 2]

Participants talked about the value of the portability of the app since employees could carry their phones with them as a “one-stop shop” (Organization 7) for information and support that they can access at any time. In terms of features, some talked about how they preferred to access the online resources; others liked the wellness check as a quick and easy tool to track change over time. It was noted that the opportunity to text, as opposed to phoning someone, was important.

*I definitely can see why the chat function would probably be-- it’s easy, accessible. People chat a lot now as opposed to picking up a phone. Like, we’re not as much a phone culture as we used to be.*

[Organization 6]

Another valued feature was the opportunity to access anonymous, private connections to information and peer support.

*There’s anonymity there. You can go and talk to someone, look at resources, set goals for yourself that nobody else is privy to. You can listen to what you want to help build your pocket of resilience*

[Organization 7]

In addition, several participants talked about the value of being able to browse the biographies of the peer support providers and choose whom and when to reach out. Overall, the opportunity to choose when and how to use the app features was noted as a benefit.

The final key facilitator relates to the characteristics and roles of individuals in the organization (champions, leaders, and peer supporters). In particular, many of the organizational champions played an active role in reaching out to their colleagues to promote the app, adopting a range of contextually relevant communication approaches. Several champions also noted the important role of supportive leaders:

*I think those types of [supportive] messages coming from leaders carry a lot of weight, you know, and especially around sensitive topics, such as mental health and self-care.*

[Organization 1]

In addition to the leaders, access to a network of trained peer support providers both within and outside the organization was noted to play a key role in facilitating engagement. This was particularly relevant for employees who did not have access to other supports. One organizational champion explained that not everyone has access to employee assistance programs (EAPs) and not all of them feel safe going to EAP. “And some of our lowest-paid workers, because they’re part-time and casual, don’t have access to EAP. The very people who need it the most” (Organization 4). The app for frontline workers therefore provided an opportunity to link with a trusted peer who had an understanding of their unique situation, filling an important gap in workplace supports.

#### Challenges or Barriers to Engagement

Despite the recognized need for mental health support and the potential for the Beyond Silence platform to meet this need, we heard about a number of barriers to adoption of the technology, many of which were related to the inner organizational context. These included cultural factors, such as workplace attitudes toward mental health and help-seeking, and structural challenges, including availability of time and space to download and use the app.

The workplace culture was identified as a challenge in many organizations in terms of trust and openness to seeking support. In some cases, we heard that there was a culture of stoicism and an expectation to manage the stress of the work.

*Based on some preliminary conversations that I’ve had with our nursing group is that they … are more inclined to, kind of assume sort of a stoicism, like, you know, "This is sort of part of the job, part of the role. … and it is the responsibility of those that are in this role to be able to deal with that.*

[Organization 1]

As outlined, many health care workers internalize the belief that their role is to provide support rather than seek it. This self-perception can hinder both the recognition and acknowledgment of personal mental health needs. Participants described a tendency among staff to often “avoid or ignore symptoms” (Organization 2A) and may not recognize a situation when the app might be valuable. Persistent stigma surrounding mental health further compounded this challenge, serving as a barrier to reaching out.

In addition to stigma, participants talked about skepticism and lack of trust, which interfered with uptake of any initiative or reaching out to others. Some explained how trust has been broken in the aftermath of COVID-19, and/or how people are tired and don’t see how things will change. One participant talked about the skepticism, with employees asking, *“*How is this gonna help me when the situation is not changing?*”* (Organization 6). They noted high levels of fatigue and questioned the value of any new initiative. Another participant talked about the overall persona of a subgroup of laboratory workers who “trust no one.” They explained that

*they’re highly skeptical. It makes them awesome at what they do. It also makes them sometimes not the easiest to work with because we trust nobody. So sharing things might be too much vulnerability for them*

[Organization 2]

It was recognized that some employees may be more receptive than others to technology, or any new initiative. One participant explained that some are “just against whatever new thing that was going to happen*”* (Organization 3)*,* and another explained that,

*there’s people who love technology and people who don’t. There’s people who really bring their issues to the team, and then there’s people who are extremely private and, you know, won’t want to be engaged in this at all*

[Organization 5]

Although some may be more receptive than others, overcoming the reluctance of frontline workers was noted to be a significant challenge both within and across organizations.

Another barrier noted by many participants was linked to the infrastructure of work. Some could not use their phones at work, had unstable internet connections, or did not have the time or private space to make a call. Given that many are short-staffed at work, they explained that there is no time even to download the app, let alone reach out for support. As one participant noted, “If it takes more than three clicks to install, then forget it. … They have so many other priorities” (Organization 5). There were many comments about the lack of time to access the app during work hours, explaining that they think “I don’t have time now, I’ll do it later,” but then later never comes (Organization 6).

Overall, analysis of the forces shaping app engagement revealed that despite perceiving the app as a valued tool that complements existing services and supports, there was a disconnect in terms of actual uptake. As one champion noted,

*“They see it. They value it. They say they like it. They recognize they need it moving forward. However… when they’re asked to opt into those types of services, uptake is almost always super low, lower than what we would expect given how much they say they value it, how much they say they like it”*

[Organization 2]

This sentiment was echoed by another participant,

*Some people said they weren’t sure why they would use it. … [others said] ‘Oh, this is great. This is a great idea.’ But they weren’t at the stage where they needed it yet. It was just at the curiosity stage*

[Organization 6]

These comments underscore the challenges of engagement and suggest that transitioning from initial interest to adoption may take time. They also reflect a perceived lack of an immediate or urgent need to use the new technology.

#### Implementation Strategies to Promote Engagement

The implementation process was an important factor that shaped uptake of the technology. Implementation included communication about the app to facilitate awareness and inspire employee engagement. This process looked a little different in each organization given their established communication channels and resources. Common engagement methods included targeted email campaigns, in-person launch events, and dissemination of printed materials such as posters, wallet cards, and flyers, and distribution of promotional materials (pens and water bottles) to enhance visibility and encourage uptake.

In all organizations, champions were asked to send email messages to employees inviting them to participate in the research study, with a brief description of the project and features of the app, a poster that provided a QR code to learn more about and download the app, and a unique organizational code to open the app. In 4 of the 7 organizations, information was posted in monthly or bimonthly staff newsletters and/or on the staff wellness section of their intranet. In general, more passive strategies (eg, email and newsletters) were seen as more efficient but not as effective in promoting engagement. As one champion noted, “I don’t think blasting emails works; our staff are always in transit” (Organization 4). Another champion talked about information overload, “[I] suspect people are just inundated with information hanging on the bulletin boards. So, it probably, unfortunately, got overlooked*”* (Organization 5).

In contrast, in-person launch events were reported to be much more effective. In six of the organizations, the materials were distributed at on-site launch events where staff could get assistance downloading the app (if needed) and ask questions about the project and the technology. The highest download rate (45%) was in a small organization where employees in a staff meeting were engaged in a structured engagement activity (20‐30 minutes), to not only download the app, but to engage with different features. In 2 of the multisite hospitals, a brief visit was scheduled at multiple sites where staff were invited to meet the team and engage with the technology. Although it was noted that in-person promotion was effective, it can also be difficult to achieve, *“*the challenge... is trying to just find people... and get in front of them for, for five minutes in, in a face-to-face*”* (Organization 1). In some cases, staff worked shifts or across different sites, so it was difficult to find a time and place to reach everyone. Not everyone was able to engage in the on-site launches due to the demands of their work; therefore, the in-person sessions only reached a small fraction of employees, particularly in larger sites. In other cases, staff worked primarily offsite so it was not possible to do an on-site launch.

In addition to the format for information sharing, the people providing the messaging had an important impact on uptake of the technology. Champions in this study came from a range of departments and levels within their respective organizations, varying from frontline supervisors to senior directors to health and wellness leads. Implementation success was often tied to a champion’s ability to leverage established networks and tailor outreach in ways that aligned with the organization’s structure. Endorsement from leadership was noted to be important, *“*leadership involvement made all the difference; when supervisors talked about the app at team meetings, it sent a message that this was important*”* (Organization 6). While top-down endorsement was valuable, champions also reflected on the potential drawbacks of delivery through formal leadership structures, particularly if staff did not trust management. As one champion noted:

*So, I think we were very well-intentioned to have our... delivery of this app come from management so that staff understood that it was valued and important. However, the double-edge of that is then perhaps the buy-in isn’t as great if folks are struggling with anything to do with the agency. So, they may see it as, ‘Here’s yet another thing my employer is trying to do, and I’m already frustrated with them. Why would I engage?’*

[Organization 5]

The same participant described the importance of trusted peers as an alternative to messaging from leaders.

*It doesn’t necessarily have to be the most extroverted person. It doesn’t even have to be anybody in any kind of formal leadership position. It just has to be somebody whose staff are willing to look to and hear them when they speak. And I think that would create better buy-in.*

[Organization 5]

The person delivering the information was therefore considered to be just as important as the nature of the message.

The other important lesson in implementation was the importance of ongoing, strategic communication to sustain interest and engagement over time. The organization with the lowest level of engagement sent an initial message but did not engage in proactive follow-up. Regular updates, transparent communication, and collaborative decision-making processes were important. Sending “push notifications” through the app itself regarding new content, or reminders to reach out was noted to be a valuable feature of the app. It was noted that “every time we push something out, you get a little [spark] in behavior*”* (Organization 2). One of the most impactful strategies seemed to be personal follow-up. As one champion noted:

*it’s really me walking around and doing check-ins. And I think it’s the champions keeping it front and center … us having it on our iPads and showing them [in peer support drop-in sessions]*

[Organization 3]

Champions talked about natural time points when a reminder about the app was effective, explaining that a critical incident in the organization would be an ideal time to remind staff about the resources available to them.

## Discussion

### Principal Findings

The study findings point to an interesting paradox regarding adoption of the Beyond Silence platform. On the one hand, we heard many positive comments about the need to support frontline health care workers and how the app met many needs for a customized, confidential tool to increase access to relevant information and peer support. On the other hand, the overall app use was relatively low (download rate of <10% in 5 of 7 organizations; average of just over 3 opens per user), which was attributed to a range of barriers to engagement.

Theories about technology adoption can shed light on the findings related to app engagement. The technology acceptance model (TAM), for example, focuses on perceived usefulness and perceived ease of use, and how these impact intention to use the technology [23]. In our study, organizational champions noted that the app was easy to use and that it was very useful in enhancing their current employee support initiatives. These beliefs, however, did not necessarily translate to frontline workers. Ease of use for frontline workers was complicated by poor internet connections in some organizations or inability to use their phones at work, and by lack of time and private space to download and use the app. Furthermore, frontline health care providers may not see the usefulness of the app given that they often ignore their own support needs in their effort to help others, and by their skepticism, lack of energy, and time to invest in new initiatives. As such, they may be more reluctant to use a digital platform to access formal peer support given that it represents a departure from simple, informal interactions with peers and requires time and effort to learn something new.

The unified theory of acceptance and use of technology (UTAUT) is another model, which builds on the factors outlined in the TAM model to expand understanding of forces influencing adoption. In addition to the focus on effort expectancy (perceptions re: ease of use) and performance expectancy (the belief that using the app will increase personal productivity), this model sheds light on the important role of social influence (beliefs that key people expect them to use the app) and facilitating conditions (belief that the necessary organizational and technical infrastructure exists to support usage) on intention to use and actual use of technology [24]. In our study, there were differences between organizations regarding both social influence and facilitating conditions. In the smallest organization, which had the highest proportion of downloads (45% in Organization 5), the organizational champion had many personal connections with their colleagues (social influence), and they set aside time during a staff meeting for employees to learn about the app and try out the different features (facilitating conditions), thereby increasing initial employee engagement. In contrast, the large professional organization with the lowest proportion of downloads (2% in Organization 2) had limited personal connection with members since members worked in diverse organizations across the country. Furthermore, this organization did not have the infrastructure to actively invest in supporting app usage, using largely passive means of communication about the opportunity to participate (initial but no follow-up emails and occasional posts about the app in the electronic membership newsletter). These implementation conditions at an organizational level with limited contact and few active engagement opportunities clearly had an impact on employee engagement.

Finally, the framework outlining NASSS of health and care technology provides additional insights to consider given that the Beyond Silence platform represents a complex intervention that is implemented at an organizational level [14]. According to the NASSS framework, there are 6 main sources of complexity that need to be considered in understanding technology implementation and uptake, namely the condition, the technology, the value proposition, the adopter system (eg, staff and patients), organization implementation, and the wider (institutional and societal) context. In the case of the Beyond Silence platform, mental health “conditions” can be considered complex in that they are often insidious, unpredictable, and stigmatized, and therefore can be difficult to identify and manage. Health care workers may fail to perceive or acknowledge their own psychological distress and often avoid seeking support due to the stigma associated with mental health issues [21,26]. The technology itself was noted to be valuable, but it is available on an “as needed” basis with no requirement for initial or ongoing use. As noted in a systematic review of eHealth interventions with health care professionals, smartphone apps have the advantage of convenience, but they may be either too flexible or too subtle to prompt regular engagement [14]. Another area of complexity relates to the value proposition for frontline workers. Although there was general buy-in from leaders, the value was not necessarily evident for frontline workers given the time, energy, and trust required to adopt something new. Organization implementation was also complex given the challenges in connecting with workers who may work different shifts, across different sites, and have little time available to download or use new technology. Understanding and addressing these dimensions of complexity is critical to creating an effective implementation plan.

The insights provided by these technology implementation theories reveal that low adoption rates may not be a failure of the technology itself, but rather a failure of implementation. There is a growing body of literature on e-mental health interventions in the workplace, including several systematic reviews [27,28], and one umbrella review of 14 review articles published between 2014 and 2023 [13]. The review studies explore a range of web-based and app-based tools, with many rooted in principles of cognitive behavioral therapy (CBT) and mindfulness, including studies that focus specifically on health care workers. Overall, there appears to be a positive impact of these tools on reducing mental distress and improving employee mental health, with a clear value proposition related to accessible, customizable tools that can expand the reach of workplace mental health initiatives [6,13,28]. These studies underscore the value of the technology itself in the context of the workplace.

A notable gap in the literature, however, is in examining implementation processes that shape uptake and impact. Studies of digital mental health interventions note variability in engagement across studies, with a paucity of consistent data to track adoption and engagement, making it difficult to conduct comparisons across different types of technology innovations and workplace contexts [29,30]. As such, this study contributes important data regarding implementation of an app-based peer support intervention, comparing implementation across a range of organizational contexts in the health care sector. The focus on an upstream mHealth intervention, rooted in self-management and peer support, is also unique, given that many studies focus on therapeutic apps (cognitive behavioral therapy and mindfulness).

### Recommendations

Based on analysis of the key facilitators and barriers noted in this study, as well as principles of implementation science theory and practice noted above, there are several recommendations to consider in order to optimize future uptake of workplace mental health apps such as the Beyond Silence platform within the context of health care workplaces. The recommendations are also based on a review of several implementation checklists and guidelines regarding strategies to foster implementation [31], including the Expert Recommendations for Implementing Change (ERIC) [32] and the European Platform to Promote Wellbeing and Health in the workplace (EMPOWER) checklist, which specifically examines strategies to foster adoption of occupational e-mental health interventions [33].

#### Evaluate Organizational Readiness for Implementation

The concept of organizational readiness to change, as theorized by Weiner [34], involves a shared resolve among organizational members (health care workers in this case) to implement a change (adoption of the Beyond Silence platform), and a shared belief in their individual and collective capability to do so. As noted in the TAM and UTAUT models, shared beliefs about the value of the app and how easy it is to use are important prerequisites to acceptance and actual use [35,36]. Readiness can be different at an individual, group, program, or organizational level [34]. The findings of our study, for example, revealed that organizational leaders appeared to be more committed and confident in adopting the Beyond Silence technology when compared to frontline workers. The degree to which they were committed to building a psychologically safe work environment beyond technology implementation; however, was not evaluated. Leadership commitment is consistently noted to be an important determinant of implementation success; trusted frontline managers can be influential, underscoring the importance of relational trust in driving intervention uptake [37]. Less is known, however, about the commitment of frontline workers. Even some programs within multisite organizations in our study were more engaged than others. Taking time to note dimensions of readiness and as well as potential sources of resistance is important in developing an implementation plan. Establishing the infrastructure to support implementation (eg, champions for change, clear, and sustained communication strategies) as well as opportunities to build collective commitment are critical to success.

#### Address Mental Health Literacy and Stigma

Beyond Silence is a platform for accessing information and support, but if people don’t recognize when and how to reach out, and/or have concerns about the stigma associated with seeking help, they may not see the need and be reluctant to use the technology. It is therefore important to build awareness such as education on burnout, moral injury, and signs of anxiety or depression, as well as access to self-screening tools (like the ones available in the app). Personal stories about seeking help from respected colleagues can be effective to reduce stigma and build a culture of proactive outreach [21,38], as is encouragement from managers and peers regarding the value of peer and professional support. We have started to develop “use case” examples for when and how to use the app, with strategies to emphasize access, noting that “help is just a click away.” To address stigma, it is also important to emphasize the privacy and anonymity features of the app and the peer support outreach.

#### Generate a Communication and Implementation Plan That Includes Multimodal Strategies to Reach a Range of Employees

Identifying who might be credible, trusted champions for change, including both leaders and frontline influencers, is a critical step since a range of voices at different levels of the organization is needed to inspire change [33]. In terms of messaging, passive strategies such as email and posters are an efficient way to reach people but may be overlooked given the many competing demands for attention. The most effective strategies involved setting aside time and space to download the app, try out features, and ask questions. Time has been noted as an influential determinant of app use in the implementation literature and one that can compromise adoption and sustainability [39]. Addressing time barriers therefore requires selecting or tailoring strategies that reduce the time burden and align with existing workflows. Implementation efforts should therefore incorporate structured assessments of time availability early in the planning process and prioritize low-effort, high-impact adaptations that facilitate integration into routine practice. Consider introducing the app at key moments (eg, new staff orientation, morning huddles, or following difficult shifts). Since staff turnover can be high in some health care settings, introducing the app as part of new employee orientation can be an effective way to embed it into organizational communication strategies. There is considerable evidence that initial and ongoing communication is also needed to sustain engagement over time [33,39]. Consider how and when to promote during the year (eg, mental health week or prior to the holiday season, which can be difficult and isolating for some employees). Without ongoing reinforcement and integration into regular workflows, engagement may decline over time [31].

#### Consider Technology Requirements, Including mHealth Access and Digital Literacy

Digital e-mental health interventions can be valuable tools to increase access to information and support, but varying levels of comfort with technology can impact adoption [33,39]. Champions in our study noted generational differences in digital literacy; therefore, it may be important to host hands-on/experiential practice sessions with employees to not only download the app but also to explore the different features. Virtual onboarding sessions, question and answer (Q&A) sessions, tutorials, frequently asked questions (FAQs; available in the app), and a contact for technology troubleshooting are important strategies to address questions that may arise over time [33]. It should be noted that some of the technology issues noted in our study were related more to the infrastructure of work, from unreliable internet to a lack of time or private space to access support. This may require organization-level interventions to build time and space for support.

#### Embed the App Platform Into Existing Organizational Policies and Practices

It takes time to introduce new ways of thinking about peer support and to build awareness, trust, and new habits regarding technology as a tool to access information and support [39]. There are many reports of the importance of social support in the context of health care [3,40], but this is often informal, and the idea of reaching out through an app may take time to build awareness and comfort with what peer support is and how it can help. Integrating the app platform into the workplace requires clear communication on how it fits within an overall model of care [33,41]. There is no “one size fits all” approach since every person is different in terms of their needs and preferences for accessing support; there may be generational differences in digital literacy and openness to accessing support, as well as different levels of need for peer support. The Beyond Silence platform was designed to promote early intervention and support but could also be another tool in the toolbox to support new employees, to facilitate connection with professional supports, or to facilitate reintegration following a workplace absence. There is currently no protocol for using the app; it is available on an “as needed” basis, but the platform is designed to easily add new content, depending upon what might be relevant to the organization. Co-developing a vision for the peer support technology within existing initiatives and working with intended users to co-create the work routines that the technology is intended to support are important strategies to optimize uptake [41]. Depending on the goal of the organization, it may not be realistic to expect all employees to use the app on a regular basis; in some cases, simply ensuring it is available when needed may be sufficient.

### Study Strengths, Limitations, and Future Work

This study provides an early examination of mental health app implementation across a diverse range of health care organizations, informed by the CFIR framework and consideration of a range of technology adoption models. There are, however, several limitations, which constrain the strength and generalizability of the findings. A 4-month implementation period provided an opportunity to observe initial short-term patterns, but may not have been sufficient to assess longer-term integration into organizational culture. The follow-up period revealed the challenges associated with maintaining long-term engagement, highlighting the importance of iterative refinement and continued evaluation. Studies of EAP use, for example, report low annual usage rates but significantly higher uptake over a 5-year period, reflecting the time required for integration into organizational culture and workflows [42]. These findings underscore the importance of examining cumulative use over time, with at least a 6‐18 month implementation period. Future research should explore extended longitudinal studies to assess delayed impacts and long-term behavioral changes. As such, the present findings should be interpreted as preliminary, and future studies should use longer observation periods to capture delayed adoption and sustained behavioral change.

The sample of 7 participating organizations also limits generalizability. Although they represented diversity in size, function, and location, all were situated within the Canadian health care system and represented a time when formal peer support and technology to support workplace health were relatively new and a time when the health care system was still recovering from the impact of the pandemic. International contexts may vary in terms of policy environments, workplace cultures, and the maturity of peer support infrastructures.

Data were only collected from organizational champions regarding why they thought that employees downloaded and engaged with (or did not engage with) the app. As nonadopters represented a significant proportion of eligible staff, their perspectives, barriers, and decision-making processes are unknown. This limits understanding of overall receptivity, organizational fit, and determinants of uptake. In addition, the absence of a comparison group restricts the ability to infer causal relationships between implementation strategies and observed outcomes.

The focus of the study was on implementation but did not track mental health or workplace outcomes of the intervention. Future research should incorporate outcome evaluation alongside implementation assessment, include comparison groups, extend follow-up periods, and gather data from both adopters and nonadopters to produce more conclusive evidence regarding effectiveness, reach, and generalizability. There is also a need to track the cost-effectiveness of this intervention approach to help employers to reflect on the value of this type of digital support intervention in their workplace context.

### Conclusion

This study provides important insights regarding implementation of the Beyond Silence peer support platform for health care workers. The findings point to a relatively slow adoption of the technology, attributed in part to the stigma and silence surrounding mental health issues, to competing demands on attention and time, and challenges with initial and ongoing engagement of frontline workers. At the same time, the findings also point to strong mission alignment for organizations that are looking for tools to support their frontline workers, and strong support for the opportunity to access high-quality customized information and resources for health care providers and to access tools to facilitate anonymous connections to trained peer supporters. We argue that understanding and addressing issues related to implementation is a necessary precondition to optimize impacts on mental health and workplace outcomes. Inspiring frontline workers to take the time and energy to access resources and peer support via the app requires reflection on potential barriers to change, and innovative approaches to build trust and inspire new ways of responding to mental health issues. Longitudinal research is needed since time is needed to study integration of the technology into workplace policies and practices.

## Abbreviations

CBT: cognitive behavioral therapy
CFIR: Consolidated Framework for Implementation Research
CIHR: Canadian Institute of Health Research
EAP: employee assistance program
EMPOWER: European Platform to Promote Wellbeing and Health in the Workplace
ERIC: Expert Recommendations for Implementing Change
FAQ: frequently asked questions
HiREB: Hamilton Integrated Research Ethics Board
mHealth: mobile health
NASSS: Nonadoption, Abandonment, Scale-up, Spread, and Sustainability
PHAC: Public Health Agency of Canada
PSW: personal support worker
PTSD: posttraumatic stress disorder
Q&A: questions and answers
TAM: technology acceptance model
UTAUT: unified theory of acceptance and use of technology
